# A 3‐miRNA signature predicts survival of patients with hypopharyngeal squamous cell carcinoma after post‐operative radiotherapy

**DOI:** 10.1111/jcmm.14702

**Published:** 2019-10-02

**Authors:** Xinbo Xu, Zhongming Lu, Neil Gross, Guojun Li, Fenghua Zhang, Dapeng Lei, Xinliang Pan

**Affiliations:** ^1^ Department of Otolaryngology Qilu Hospital of Shandong University Jinan China; ^2^ NHC Key Laboratory of Otorhinolaryngology Shandong University Jinan China; ^3^ Department of Otolaryngology‐Head and Neck Surgery Guangdong Provincial People's Hospital and Guangdong Academy of Medical Sciences Guangzhou China; ^4^ Department of Head and Neck Surgery The University of Texas MD Anderson Cancer Center Houston Texas; ^5^ Department of Epidemiology The University of Texas MD Anderson Cancer Center Houston Texas; ^6^ Thyroid and Breast Surgery Department Hebei General Hospital Shijiazhuang China

**Keywords:** biomarker, hypopharyngeal cancer, miRNA, score, signature, survival

## Abstract

Since the prognosis of hypopharyngeal squamous cell carcinoma (HSCC) remains poor, identification of miRNA as a potential prognostic biomarker for HSCC may help improve personalized therapy. In the 2 cohorts with a total of 511 patients with HSCC (discovery: N = 372 and validation: N = 139) after post‐operative radiotherapy, we used miRNA microarray and qRT‐PCR to screen out the significant miRNAs which might predict survival. Associations of miRNAs and the signature score of these miRNAs with survival were performed by Kaplan‐Meier survival analysis and multivariate Cox hazard model. Among 9 candidate, miRNAs, miR‐200a‐3p, miR‐30b‐5p, miR‐3161, miR‐3605‐5p, miR‐378b and miR‐4451 were up‐regulated, while miR‐200c‐3p, miR‐429 and miR‐4701 were down‐regulated after validation. Moreover, the patients with high expression of miR‐200a‐3p, miR‐30b‐5p and miR‐4451 had significantly worse overall survival (OS) and disease‐specific survival (DSS) than did those with low expression (log‐rank *P* < .05). Patients with a high‐risk score had significant worse OS and DSS than those with low‐risk score. Finally, after adjusting for other important prognostic confounders, patients with high expression of miR‐200a‐3p, miR‐30b‐5p and miR‐4451 had significantly high risk of overall death and death owing to HSCC and patients with a high‐risk score has approximately 2‐fold increased risk in overall death and death owing to HSCC compared with those with a low‐risk score. These findings indicated that the 3‐miRNA‐based signature may be a novel independent prognostic biomarker for patients given surgery and post‐operative radiotherapy, supporting that these miRNAs may jointly predict survival of HSCC.

## 1 INTRODUCTION

Hypopharyngeal squamous cell carcinoma (HSCC) is an aggressive malignancy among head and neck squamous cell cancers. It accounts for approximately 4% of the total cases of head and neck cancers.^1^ Although the incidence is approximately 0.7‐0.95 per million person‐years with a declining trend over the past 4 decades,^1^, ^2^, ^3^ an estimated 3000 new cases are diagnosed with HSCC annually in the United States.^4^ The combined treatments of surgery, radiotherapy and chemotherapy have been applied, while the overall prognosis of patients with HSCC remains poor because of the high rate of local recurrence and nodal metastasis. The average 5‐year overall survival has improved only from 37.5% to 41.3% between before 1990 and after 1990 according to the SEER data.^5^

Many proteins have been selected for prognostic biomarkers of HSCC, such as the truncating mutation of TP53, CD105 expression, CD98, and Oct4 and osteopontin in tumours,^6^ and however, the proteins or genome DNA has the tendency of degradation, which limits their clinical application. Thus, a molecule with more stable characteristics is more suitable to be a candidate biomarker. miRNAs are small non‐coding RNAs with potent regulatory functions in cell growth, proliferation and apoptosis.^7^ It has been demonstrated that expression levels of miRNAs which function as tumour suppressors and oncogenes are dysregulated in different cancers.^8^ A growing body of evidence indicates that miRNA profiles are associated with particular cancers and could serve as potential biomarkers for tumour diagnosis and prognosis.^9^ Unlike the proteins, miRNAs are quite stable in clinical archived samples such as serum samples or formalin‐fixed paraffin‐embedded (FFPE) tissue specimens. In addition, miRNAs could be detected and their degradation of miRNA expression was very minimal in various types of cancer tissue samples after a long‐term storage.^10^ Thus, human miRNAs from FFPE tissue specimens are highly stable, and FFPE tissue‐based miRNAs hold great promise as ideal candidate biomarkers for diagnosis and prognosis of HSCC in clinical practice.

Our previous study showed that the expression level of miR‐4451 in patients with HSCC was significantly associated with prognosis.^11^ To improve the efficiency and accuracy of prediction of prognosis in HSCC, we developed a risk model based on these three‐miRNA signature for improved prognostic prediction of HSCC. The 3‐miRNA signature allows us to assess the combined effects of a panel of miRNA expression profiles that act in the same pathway. Evaluating such combined effects may amplify the associations of individual miRNA expression with the prognosis of HSCC.

## 2 MATERIALS AND METHOD

### 2.1 Patients and samples

The patients included in this study were diagnosed with HSCC pathologically and underwent surgery with post‐operative radiotherapy at the Department of Otolaryngology, Qilu Hospital of Shandong University from January 2012 to January 2016. The patients enrolled from the Jinan Branch of Department were assigned into a discovery cohort, whereas the patients of Qingdao Branch into an independent validation cohort. None of the patients received any chemotherapy. If the patients had no medical records, low RNA samples output for miRNA or lost during the post‐operative follow‐up, they were excluded from this study. All the patients were well informed when admitted and signed the consent forms. This study was approved by the Medical Ethical Committee of the Qilu Hospital (2017061).

The epidemiological variables, including age, sex, exposure to cigarettes and alcohol, and the clinical variables, including primary sites, TNM staging and pathological grade, were reviewed from the patients’ medical records. Two senior physicians determined the TNM stage based on pre‐operative examination, surgical and pathological report according to the American Joint Committee on Cancer Staging Manual (the 8th Ed.). The current vital status (death or alive) was recorded by retrospective interview in January 2019 through phone call or by mail. The end outcomes of interest in this study were overall survival (OS) and disease‐specific survival (DSS). OS was defined as the time (months) from the date of first treatment to death from any reasons or date of last follow‐up. Participants who were alive at the end of the study period or lost to follow‐up were considered censored. DSS was defined as the time from the date of first treatment to death from disease or date of last follow‐up.

The FFPE blocks of patients were retrieved from the Department of Pathology, Qilu Hospital of Shandong University. All the FFPE tissue specimens were harvested and stored under the standard protocol. Slices of 10 μm thick were made, and the superficial 3 slices were discarded. The two senior pathologists identified the tumour stroma in a haematoxylin‐eosin–stained slice, after the mucosal and necrotic regions of other stainless slices were removed. Depending on the size of the tumour stroma, 2‐4 sections per patients were used. After deparaffinization, the RecoverAll Total Nucleic Acid Isolation Kit, for FFPE Tissues (Ambion), was used for total RNA extraction following the manufacturer's protocol. NanoDrop ND 3.0 spectrophotometer (NanoDrop Technologies Inc) and Agilent 2100 Bioanalyzer (Agilent Technologies) were used for quantification and quality control of RNA samples.

### 2.2 High‐throughput miRNA expression profiling

An Agilent Human miRNA microarray (Release 21.0, 8*60K) platform was used for high‐throughput miRNA expression profiling in the discovery cohort. This microarray detected 2549 miRNAs from Sanger miRBase 21 with 20 replicated probes for every single miRNA. The whole cohort was divided into 2 groups simply by the vita status (dead vs alive). Each group included 4 cases. As Agilent technical support, the 100ng of total RNA of each sample was dephosphorylated, denatured and ligated with the Cy3 fluorescent label. Then, the purified samples were hybridized for 10 minutes and washed for twice. Agilent G2505C Microarray Scanner System and Agilent Feature Extraction Software (Agilent Technologies) were used to scan the microarray and extract the signal of each probe. The value of each miRNA was subtracted by the average background value and normalized using a quantile method.

Differentially expressed miRNAs were identified by two criteria: (a) the fold change (FC) of miRNA between the two groups ≥ 1.5 and *P*‐value ≤ .05 and (b) the miRBase ID of the candidate miRNA was <5000. The FC value was calculated by a formula as follows: 2^ |(average normalized expression value of death group – average normalized expression value of survival group)|. The *P*‐value was generated by unpaired *t* test. Whether the miRNA was up‐regulated or down‐regulated was determined by the average miRNA expression in the two groups.

### 2.3 qRT‐PCR analysis

After high‐throughput screening by microarray, the candidate miRNAs were verified by qRT‐PCR analysis. As previously described,^12^ the 100 ng of total RNA of each sample was polyadenylated and reversely transcripted to cDNA. Then, a quantified PCR was carried out in the Qiagen miScript system (Qiagen) on the ABI 7900HT platform (ABI). The U48 was selected as endogenous control. The Cq value was generated by SDS software 2.4 (ABI). Through data processing by Thermo Fisher Cloud (https://apps.thermofisher.com/apps/dashboard/), the Cq values were adjusted by interplate calibrator (IC), and the relative expression of candidate miRNA was calculated as 2^‐ΔCq, where ΔCq = Cq (candidate miRNA) − Cq (endogenous control).

### 2.4 miRNA signature score and survival analysis

The expression of each miRNA was categorized into two groups (1 = high expression and 0 = low expression) with a median value of ΔCq as the cut‐off point. We categorized the three significant miRNAs of interest into a new variable. Specifically, we categorized all putative risk (HRs > 1.0) of each miRNA into a new variable according to the expression of miRNA carried by an individual for each of the 3 miRNAs, while we reversed the reference group to reflect the protective effects of the miRNA expression. Therefore, according to the score of miRNA expression carried by each individual and the level of HRs linked to the expression of each individual miRNA, a risk model for miRNA signature was builded up based on association of the validated miRNA expression with survival. We categorized the individuals into the high‐risk (score = 2 or 3) and low‐risk groups (score = 0 or 1) to evaluate the collective effects of the three miRNAs on survival. The differences between epidemiological/clinical variables and the miRNA expressions/signature score were tested by Chi‐square or Fisher's exact test. The Kaplan‐Meier method was used to test whether OS and DSS were significantly associated with survival for miRNA expression, epidemiological and clinical variables. The significant variables for survival prediction suggested by the Kaplan‐Meier survival analysis were included in a multivariate Cox proportional hazards regression models for associations of survival with miRNA expression or risk score of miRNA signatures. All the procedure was performed in the Statistical Product and Service Solutions (version 23; IBM). Microsoft Excel and GraphPad Prism were used for scientific drawing. The statistical significance was set at *P* < .05, and all tests were 2‐sided.

## 3 RESULT

### 3.1 Patients’ characteristics

According to inclusion and exclusion criteria, a total of 436 HSCC patients’ medical records were reviewed in discovery cohort. Among them, 408 patients received surgery and post‐operative radiotherapy. Eleven patients were lost for follow‐up, and 8 patients without correct diagnosis were excluded. In the remaining 389 patients who had FFPE tissues available, 8 cases had massive necrosis in samples and 9 cases had low concentration of RNA output for qRT‐PCR. These patients were also excluded. Thus, a total of 372 patients were included in discovery cohort. Similarly, we used the same inclusion and exclusion criteria as in discovery cohort, and a total of 139 out of 157 patients with HSCC were included in the validation cohort.

The clinical and epidemiological variables were not significantly different between the two cohorts (Table 1). The discovery cohort contained 349 male patients (93.8%) and 23 female patients (6.2%), and the validation cohort had 129 male patients (92.8%) and 10 female patients (7.2%). The median ages of these patients at the time of diagnosis were 59 years (Mean: 59.3) in discovery cohort and 57 years in validation cohort (Mean: 58.1). A total of 73 patients (19.6%) were non‐smokers, and 96 patients (25.8%) have no history of alcohol drinking in discovery cohort, whereas there were 24 non‐smokers (17.3%) and 35 non‐drinkers (25.2%) in validation cohort. Similarly to our previous report,^12^ in both cohort there were no significant associations between OS and epidemiological/clinical variables, such as age, sex, smoking and drinking status except the T and N stage, having a high grade of T (T4) or N (N2/N3) with a worse prognosis.^12^

**Table 1:** Characteristics of epidemiological and clinical variables in two cohorts

Variables	Discovery cohort	Validation cohort	*P*
Age			
≤59	177	63	.649
>59	195	76	
Sex			
Male	349	129	.679
Female	23	10	
Smoking			
Never	73	24	.545
Ever	299	115	
Drinking			
Never	96	35	.885
Ever	276	104	
Site			
Piriform	298	110	.808
Post Wall + Post‐cricoid	74	29	
Differentiation			
Poor/Moderate	275	101	.773
Well	97	38	
T classification			
T1‐3	325	123	.731
T4	47	16	
N classification			
N0‐1	173	70	.438
N2‐3	199	69	

### 3.2 Identification of candidate miRNAs by microarray

By miRNA microarray, differentially expressed miRNAs were selected under two criteria. A total of 14 miRNAs were found to match the 1st criteria: FC ≥ 1.5 and *P*‐value ≤ .05 between the two groups. These miRNAs included miR‐200a‐3p, miR‐200c‐3p, miR‐30b‐5p, miR‐3161, miR‐3195, miR‐3605‐5p, miR‐378b, miR‐429, miR‐4451, miR‐4688, miR‐4701, miR‐5088‐5p, miR‐6808‐5p and miR‐6813‐3p. Because miRNAs with higher miRBase ID might be artefacts, we excluded miR‐5088‐5p, miR‐6808‐5p and miR‐6813‐3p. In our preliminary experiment, because we found the PCR reactions for miR‐3195 and miR‐4688 generated unsatisfactory dissociation curve and segregated strips in electrophoresis, these two miRNAs were also excluded.

**Figure 1:**
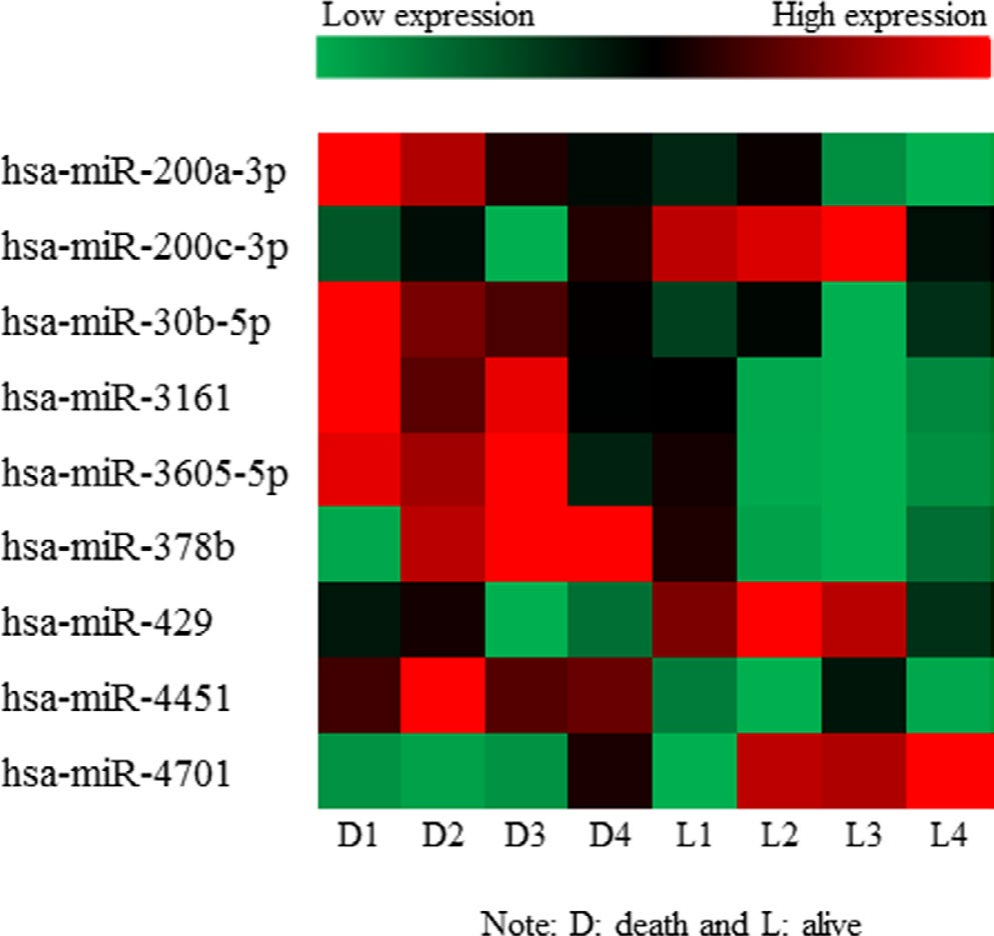
Hot map of differently expressed miRNAs between death and alive groups

Finally, totally 9 miRNAs were selected as candidate miRNAs as shown in Figure 1. Among them, 6 miRNAs (miR‐200a‐3p, miR‐30b‐5p, miR‐3161, miR‐3605‐5p, miR‐378b and miR‐4451) were up‐regulated, whereas 3 miRNAs (miR‐200c‐3p, miR‐429 and miR‐4701) were down‐regulated in death group compared with alive group. After validation of the 9 candidate miRNAs by qRT‐PCR, we performed the relative quantification of the miRNAs using U48 as endogenous control. The whole data set was calibrated by IC, and the dissociation curves and electrophoresis of the PCR reactions for 9 candidate miRNAs were checked to ensure the guaranteed PCR results. Moreover, miR‐4701 was found to have the lowest *P*‐value (*P* = .005), and the highest FC value (FC = 9.6). The whole set of raw data can be accessed in the GEO database (GSE117558).

In both cohorts, the expressions of 3 miRNAs were not associated with epidemiological variables, except the high expression of miR‐200a‐3p in smokers. In both cohorts, high expression of miR‐200a‐3p was correlated with a high grade of T (T4) or N (N2/N3), whereas high expression of miR‐30b‐5p was correlated only with a high grade of T (T4). Moreover, in the discovery cohort, there was a significant association between high expression of miR‐4451 and a high grade of T (T4) (Table 2).

**Table 2:** Distribution and associations of 3 significant miRNA expression/signature score in patients’ characteristics

Variables	Discovery cohort												Validation cohort											
	miR‐200a‐3p			miR‐30b‐5p			miR‐4451			Signature score			miR‐200a‐3p			miR‐30b‐5p			miR‐4451			Signature score		
	Low (%)	High (%)	*P*	Low (%)	High (%)	*P*	Low (%)	High (%)	*P*	Low (%)	High (%)	*P*	Low (%)	High (%)	*P*	Low (%)	High (%)	*P*	Low (%)	High (%)	*P*	Low (%)	High (%)	*P*
Age																								
≤59	94 (50.0)	83 (45.1)	.345	87 (46.8)	90 (48.4)	.755	91 (50)	86 (45.3)	.360	87 (49.2)	90 (46.2)	.563	36 (45.0)	27 (45.8)	.929	37 (46.8)	26 (43.3)	.681	34 (45.3)	29 (45.3)	.998	35 (44.9)	28 (45.9)	.904
>59	94 (50.0)	101 (54.9)		99 (53.2)	96 (51.6)		91 (50)	104 (54.7)		90 (50.8)	105 (53.8)		44 (55.0)	31 (54.2)		42 (53.2)	34 (56.7)		41 (54.7)	35 (54.7)		43 (55.1)	33 (54.1)	
Sex																								
Male	179 (95.2)	170 (92.4)	.259	172 (92.5)	177 (95.2)	.282	171 (93.7)	178 (93.7)	.913	166 (93.8)	183 (93.8)	.981	76 (95.0)	53 (89.8)	.244	73 (92.4)	56 (93.3)	.834	71 (94.7)	58 (90.6)	.358	73 (93.6)	56 (91.8)	.686
Female	9 (4.8)	14 (7.6)		14 (7.5)	9 (4.8)		11 (6.3)	12 (6.3)		11 (6.2)	12 (6.2)		4 (5.0)	6 (10.2)		6 (7.6)	4 (6.7)		4 (5.3)	6 (9.4)		5 (6.4)	5 (8.2)	
Smoking																								
Never	38 (20.2)	35 (19.0)	.772	38 (20.4)	35 (18.8)	.695	35 (19.2)	38 (20.0)	.852	36 (20.3)	37 (19.0)	.741	15 (18.8)	9 (15.3)	.590	14 (17.7)	10 (16.7)	.871	13 (17.3)	11 (17.2)	.982	15 (19.2)	9 (14.8)	.488
Ever	150 (79.8)	149 (81.0)		148 (79.6)	151 (81.2)		147 (80.8)	152 (80.0)		141 (79.7)	158 (81.0)		65 (81.3)	50 (84.7)		65 (82.3)	50 (83.3)		62 (82.7)	53 (82.8)		63 (80.8)	52 (85.2)	
Drinking																								
Never	58 (30.9)	38 (20.7)	.025*	51 (27.4)	45 (24.2)	.477	53 (29.1)	43 (22.6)	.153	55 (31.1)	41 (21.0)	.027	23 (28.8)	12 (20.3)	.259	22 (27.8)	13 (21.7)	.406	20 (26.7)	15 (23.4)	.662	24 (30.8)	11 (18.0)	.086
Ever	130 (69.1)	146 (79.3)		135 (72.6)	141 (75.8)		129 (70.9)	147 (77.4)		122 (68.9)	154 (79.0)		57 (71.3)	47 (79.7)		57 (72.2)	47 (78.3)		55 (73.3)	49 (76.6)		54 (69.2)	50 (82.0)	
Site																								
Piriform	145 (77.1)	153 (83.2)	.146	150 (80.6)	148 (79.6)	.795	149 (81.9)	149 (78.4)	.405	140 (79.1)	158 (81.0)	.403	62 (77.5)	48 (81.4)	.580	65 (82.3)	45 (75.0)	.296	62 (82.7)	48 (75.0)	.268	61 (78.2)	49 (80.3)	.760
PW + PC	43 (22.9)	31 (16.8)		36 (19.4)	38 (20.4)		33 (18.1)	41 (21.6)		37 (20.9)	37 (19.0)		18 (22.5)	11 (18.6)		14 (17.7)	15 (25.0)		13 (17.3)	16 (25.0)		17 (21.8)	12 (19.7)	
Differentiation																								
P/M	137 (72.9)	138 (75.0)	.640	136 (73.1)	139 (74.7)	.723	131 (72.0)	144 (75.8)	.403	126 (71.2)	149 (76.4)	.252	58 (72.5)	43 (72.9)	.960	59 (74.7)	42 (70.0)	.539	53 (70.7)	48 (75.0)	.568	55 (70.5)	46 (75.4)	.520
Well	51 (27.1)	46 (25.0)		50 (26.9)	47 (25.3)		51 (28.0)	46 (24.2)		51 (28.8)	46 (23.6)		22 (27.5)	16 (27.1)		20 (25.3)	18 (30.0)		22 (29.3)	16 (25.0)		23 (29.5)	15 (24.6)	
T																								
T1‐3	175 (93.1)	150 (81.5)	.001*	173 (93.0)	152 (81.7)	.001*	166 (91.2)	159 (83.7)	.029*	164 (92.7)	161 (82.6)	.003*	77 (96.3)	46 (78.0)	.001*	74 (93.7)	49 (81.7)	.028*	70 (93.3)	53 (82.8)	.053	75 (96.2)	48 (78.7)	.001*
T4	13 (6.9)	34 (18.5)		13 (7.0)	34 (18.3)		16 (8.8)	31 (16.3)		13 (7.3)	34 (17.4)		3 (3.8)	13 (22.0)		5 (6.3)	11 (18.3)		5 (6.7)	11 (17.2)		3 (3.8)	13 (21.3)	
N																								
N0‐1	97 (51.6)	76 (41.3)	.047*	93 (50.0)	80 (43.0)	.177	91 (50.0)	82 (43.2)	.186	94 (53.1)	79 (40.5)	.015*	46 (57.5)	24 (40.7)	.050*	42 (53.2)	28 (46.7)	.448	4 (58.7)	26 (40.6)	.340	47 (60.3)	23 (37.7)	.008*
N2‐3	91 (48.4)	108 (58.7)		93 (50.0)	106 (57.0)		91 (50.0)	108 (56.8)		83 (46.9)	116 (59.5)		30 (42.5)	35 (59.3)		37 (46.8)	32 (53.3)		31 (41.3)	38 (59.4)		31 (39.7)	38 (62.3)	

Note

*P*: Fisher's exact tests were used to evaluate the differences between epidemiological/clinical conditions and the miRNA expressions.

Abbreviations: P/M, poor/moderate; PC, post‐cricoid; PW, posterior wall.

a *P *≤ .05.

### 3.3 Survival analysis on candidate miRNAs

**Figure 2:**
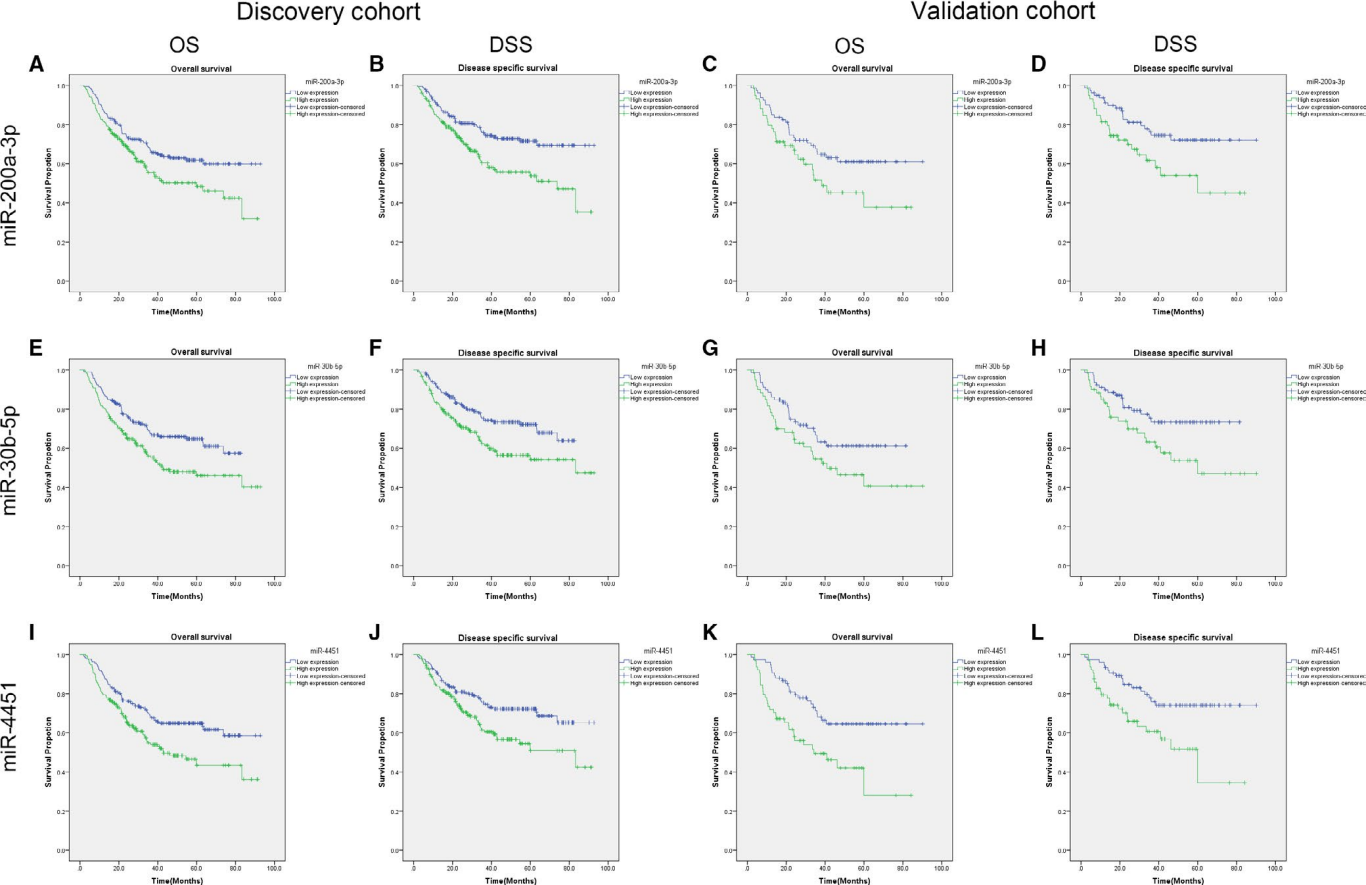
Kaplan‐Meier estimates for OS and DSS of patients in discovery/validation cohort according to the expressions of each of the 3 individual miRNAs

As shown in Table 3, in the discovery cohort, among the 9 candidate miRNAs, the average OS time in the groups with high miR‐200a‐3p, miR‐30b‐5p and miR‐4451 expression was 52.8 months (95% CI: 46.8‐58.7), 53.7 months (95% CI: 47.8‐59.5) and 52.2 months (95% CI: 46.6‐58.1), respectively, whereas the average survival time in the groups with corresponding low expression was 64.8 months (95% CI: 59.4‐70.1), 59.7 months (95% CI: 55.0‐64.3) and 65.6 months (95% CI: 60.2‐71.0), respectively. The average DSS time in the groups with high miR‐200a‐3p, miR‐30b‐5p and miR‐4451 expression was 56.7 months (95% CI: 50.5‐62.8), 59.4 months (95% CI: 53.5‐65.4) and 57.9 months (95% CI: 51.7‐64.1), respectively, whereas the average survival time in the groups with corresponding low expression was 71.7 months (95% CI: 66.6‐76.8), 64.3 months (95% CI: 59.9‐68.8) and 70.6 months (95% CI: 65.3‐75.9), respectively. The Kaplan‐Meier survival analysis showed that the patients with the high expressions of miR‐200a‐3p, miR‐30b‐5p and miR‐4451 affected both OS and DSS. The patients with low expression of miR‐200a‐3p, miR‐30b‐5p and miR‐4451 experienced a significantly better prognosis than those with corresponding high expressions shown in Figure 2. In the validation cohort, the low expressions of miR‐200a‐3p and miR‐4451 were associated with better OS and DSS, while the low expression of miR‐30b‐5p was only associated with better DSS, rather than OS. (Figure 2 and Table 3).

**Table 3:** OS and DSS (in months) of study patients in 2 cohorts with expression of 3 candidate miRNAs/signature score

miRNAs	Discovery cohort				Validation cohort			
	OS		DSS		OS		DSS	
	Mean (95% CI)	*P*	Mean (95% CI)	*P*	Mean (95% CI)	*P*	Mean (95% CI)	*P*
miR‐200a‐3p								
Low expression	64.8 (59.4‐70.1)	.012*	71.7 (66.6‐76.8)	.001*	63.1 (55.2‐70.9)	.048*	71.1 (63.6‐78.5)	.016*
High expression	52.8 (46.8‐58.7)		56.7 (50.5‐62.8)		46.9 (37.4‐56.4)		51.9 (42.0‐61.9)	
miR‐30b‐5p								
Low expression	59.7 (55.0‐64.3)	.004*	64.3 (59.9‐68.8)	.004*	57.8 (50.7‐65.1)	.068	64.8 (58.0‐71.6)	.032*
High expression	53.7 (47.8‐59.5)		59.4 (53.5‐65.4)		50.6 (40.7‐60.4)		56.1 (45.8‐66.2)	
miR‐4451								
Low expression	65.6 (60.2‐71.0)	.003*	70.6 (65.3‐75.9)	.006*	66.4 (58.6‐74.2)	.002*	72.4 (65.1‐79.8)	.005*
High expression	52.2 (46.6‐58.1)		57.9 (51.7‐64.1)		42.6 (32.9‐52.3)		48.8 (38.1‐59.6)	
Signature score								
Low risk	67.7 (62.4‐73.2)	.000*	73.0 (67.8‐78.1)	.000*	67.9 (60.3‐75.5)	.000	73.9 (66.8‐80.9)	.001*
High risk	50.7 (44.9‐56.4)		56.2 (50.3‐62.2)		41.2 (32.1‐50.4)		47.7 (37.6‐57.8)	

Note

*P*: Log‐rank test was used for differences between different subgroups.

a *P* ≤ .05.

### 3.4 Association of candidate miRNAs with risk of OS

As shown in Table 4, the univariate analysis showed that the patients with high expression of miR‐200a‐3p and miR‐30b‐5p had approximately 1.5‐ to 1.6‐fold increased risk of overall death and 1.7‐ to 1.8‐fold increased risk of death owing to the disease compared with the patients with corresponding low expression, respectively (For OS: cHR, 1.5; 95% CI, 1.1‐2.9 for miR‐200a‐3p and 1.6; 95% CI, 1.1‐2.2 for miR‐30b‐5p; for DSS: cHR, 1.8; 95% CI, 1.3‐2.6 for miR‐200a‐3p and 1.7; 95% CI, 1.2‐2.5 for miR‐30b‐5p). Similarly, the patients with high expression of miR‐4451 had approximately 1.6‐fold increased risk of overall death (cHR, 1.6; 95% CI, 1.1‐2.2) compared with those with its low expression as we previously reported.^12^ The risk of death owing to the disease was 70% higher in patients with high expression of miR‐4451 (cHR, 1.7; 95% CI, 1.2‐2.4) than those with the corresponding low expression. Furthermore, after adjustment with other important confounders in multivariable Cox hazard models including age, sex, smoking, alcohol, primary sites, differentiation, T classification and N classification, the patients with high expression of the same miRNAs had approximately 40%‐60% increased risk of overall death and death specific to the disease compared with the corresponding low expression of the 3 miRNAs (For OS: aHR, 1.4; 95% CI, 1.0‐1.9 for miR‐200a‐3p, 1.4; 95% CI, 1.0‐2.0 for miR‐30b‐5p, and 1.5; 1.0‐2.0 for miR‐4451; for DSS: aHR, 1.6; 95% CI, 1.1‐2.4 for miR‐200a‐3p, 1.5; 95% CI, 1.1‐2.3 for miR‐30b‐5p, and 1.6; 1.1‐2.3 for miR‐4451, respectively) (Table 3).

**Table 4:** Association of 3 miRNAs/signature score with OS and DSS in patients with HSCC

miRNAs/Signature score	Discovery cohort				Validation cohort			
	Univariate		Multivariable		Univariate		Multivariable	
	cHR (95%CI)	*P*	aHR (95%CI)	*P*	cHR (95%CI)	*P*	aHR (95%CI)	*P*
miR‐200a‐3p (High vs Low)								
OS	1.5 (1.1‐2.9)	.012*	1.4 (1.0‐1.9)	.037*	1.7 (1.0‐2.8)	.049*	1.3 (0.7‐2.9)	.379
DSS	1.8 (1.3‐2.6)	.001*	1.6 (1.1‐2.4)	.010*	2.1 (1.1‐3.8)	.019*	1.5 (0.8‐3.0)	.189
miR‐30b‐5p (High vs Low)								
OS	1.6 (1.1‐2.2)	.004*	1.4 (1.0‐2.0)	.026*	1.6 (0.9‐2.7)	.071	1.5 (0.7‐2.0)	.270
DSS	1.7 (1.2‐2.5)	.004*	1.5 (1.1‐2.3)	.025*	1.9 (1.0‐3.6)	.035*	1.6 (0.8‐2.9)	.155
miR‐4451 (High vs Low)								
OS	1.6 (1.1‐2.2)	.003*	1.5 (1.0‐2.0)	.023*	2.2 (1.3‐3.8)	.003*	1.8 (1.0‐3.2)	.040*
DSS	1.7 (1.2‐2.4)	.007*	1.6 (1.1‐2.3)	.022*	2.4 (1.3‐4.4)	.007*	1.8 (1.0‐3.5)	.049*
Signature score (High vs Low)								
OS	2.0 (1.4‐2.7)	.000*	1.8 (1.3‐2.5)	.001*	2.6 (1.5‐4.5)	.000*	2.1 (1.2‐3.7)	.013*
DSS	2.1 (1.4‐3.1)	.000*	1.9 (1.3‐2.8)	.001*	2.8 (1.5‐5.4)	.001*	2.1 (1.1‐4.2)	.028*

a *P *≤ .05.

After validation in another independent cohort, the univariate analysis showed that the high expression of miR‐200a‐3p and miR‐4451 increased risk of overall death and death owing to the disease (For OS: cHR, 1.7; 95% CI, 1.0‐2.8 for miR‐200a‐3p and 2.2; 95% CI, 1.3‐3.8 for miR‐4451; for DSS: cHR, 2.1; 95% CI, 1.1‐3.8 for miR‐200a‐3p and 2.4; 95% CI, 1.3‐4.4 for miR‐4451), whereas the high expression of miR‐30‐5p only increased risk of death owing to the disease (cHR, 1.9; 95% CI, 1.0‐3.6) but not for overall death. Moreover, after adjusting with other important prognostic confounders, we found that only the significant association remained for miR‐4451 only (For OS: aHR, 0.8; 95% CI, 1.0‐3.2; for DSS: aHR, 1.8; 95% CI, 1.0‐3.5), but not for miR‐200a‐3p and miR‐30‐5p (data not shown). Such a discordancy between the two cohorts could be partially due to the sample size (the sample size was relatively small in the validation cohort).

### 3.5 Effect of a signature score of miRNAs on risk of OS

The HRs of miR‐200a‐3p, miR‐30b‐5p or miR‐4451 were all >1, so all the 3 miRNAs were risky to OS and DSS. As we described in the method, high expressions of miRNAs were defined as a value of 1, whereas low expression as 0. By summating the values of miRNA in this signature, a patient might get a score of 0‐3. The higher score meant a patient expressed more risky miRNAs.

**Figure 3:**
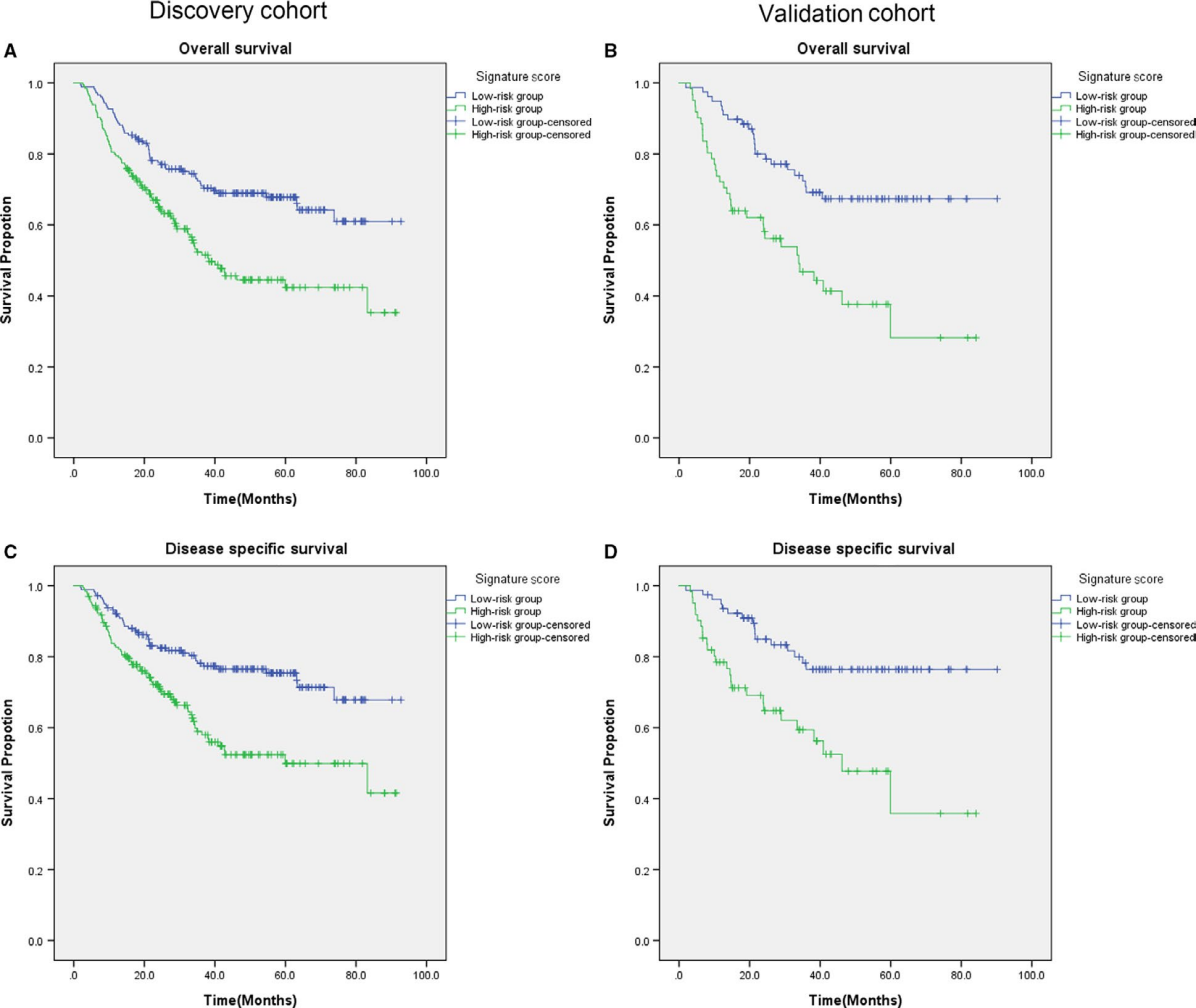
Kaplan‐Meier estimates for the OS and DSS of patients in discovery/validation cohort according to the 3‐miRNA signature score. The high‐risk group included patients with 2‐3 risk score, and the low‐risk group included patients with 0‐1 risk score

According to the risk signature score, we divided the patients into a low‐risk group (score = 0 or 1) and a high‐risk group (score = 2 or 3). In the discovery cohort, the average OS and DSS of patients with the low‐risk/high‐risk group were 67.8 months (95% CI: 62.4‐73.1)/50.7 months (95% CI: 44.9‐56.4) and 73.0 months (95% CI: 67.8‐78.1)/56.2 months (95% CI: 50.3‐62.2) (Table 3). As shown in Figure 3, the patients with high‐risk score had significantly worse OS and DSS than those with low‐risk score (log‐rank: *P*_OS_ < 0.001 and *P*_DSS_ < .001). Furthermore, both the univariate and multivariable analyses showed the patients with high‐risk score of the 3 miRNAs had approximately 2‐fold significantly increased risk of overall death and death due to the disease compared with the patients with a low‐risk score (For OS: cHR, 2.0; 95% CI, 1.4‐2.7; for DSS: cHR, 2.1; 95% CI, 1.4‐3.1 and for OS: aHR, 1.8; 95% CI, 1.3‐2.5; for DSS: aHR, 1.9; 95% CI, 1.3‐2.8) (Table 4). Similarly, as in the discovery cohort, after validation in the validation cohort, we found that the OS and DSS were shorter in high‐risk group than in low‐risk group, while the high score of miRNAs also increased risk of overall death and death due to the disease compared with the patients with a low‐risk score.

### 3.6 Diagnostic value of miRNA signature score for prognosis prediction

**Figure 4:**
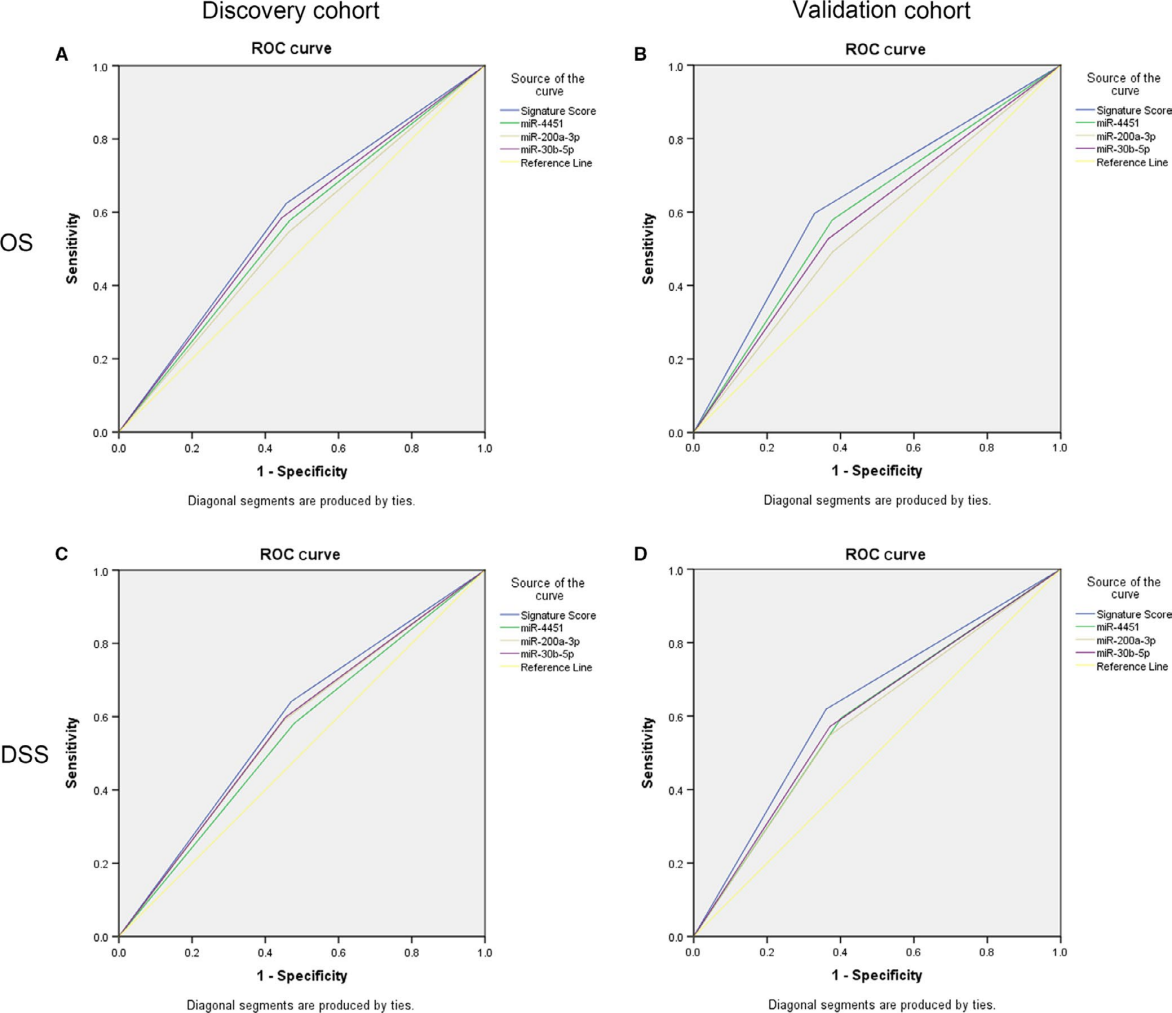
ROC curve of 3 significant miRNA expression/signature score for predicting prognosis of OS and DSS

As shown in Figure 4 and Table 5, the area under the ROC curve (AUC) was applied to compare the prognostic accuracy between models with different miRNAs. In the discovery cohort, the signature score (AUC: OS, 0.583; DSS, 0.585) showed more reliable prognostic effect than the other 3 miRNAs independently for both OS and DSS. The sensitivity and specificity of model with signature score was 0.624 and 0.543 when predicting OS, and 0.641 and 0.529 when predicting DSS. Validated in another cohort, the signature score also showed a similarly reliable prognostic effect (AUC: OS, 0.634; DSS, 0.629), and the sensitivity and specificity was 0.596 and 0.671 when predicting OS, and 0.619 and 0.639 when predicting DSS. The ROC curves suggested that the 3‐miRNA‐based signature score can serve as biomarkers for predicting prognosis of HSCC.

**Table 5:** AUG of ROC analysis for prognosis of OS and DSS

miRNAs	Discovery cohort				Validation cohort			
	OS		DSS		OS		DSS	
	AUC (95% CI)	*P*	AUC (95% CI)	*P*	AUC (95% CI)	*P*	AUC (95% CI)	*P*
miR‐200a‐3p	0.541 (0.481‐0.601)	.181	0.569 (0.507‐0.632)	.032*	0.557 (0.459‐0.654)	.257	0.588 (0.484‐0.692)	.099
miR‐30b‐5p	0.570 (0.511‐0.629)	.022*	0.572 (0.509‐0.634)	.026*	0.580 (0.483‐0.677)	.108	0.600 (0.497‐0.703)	.061
miR‐4451	0.555 (0.496‐0.615)	.070*	0.551 (0.489‐0.614)	.111	0.600 (0.504‐0.697)	.044*	0.597 (0.494‐0.700)	.071
Signature score	0.583 (0.524‐0.642)	.006*	0.585 (0.523‐0.647)	.008*	0.634 (0.539‐0.728)	.007*	0.629 (0.528‐0.731)	.016*

a *P* ≤ .05.

## 4 DISCUSSION

In this study, we have identified a three‐miRNA signature that was significantly associated with OS and DSS of patients with HSCC undergoing surgery and radiotherapy. The high expression of each of these miRNAs may predict an increased risk of death, while the combined 3‐miRNA‐based signature is novel and more robust prognostic biomarkers for patients with HSCC after post‐operative radiotherapy, with a higher risk score having worse survival.

Squamous cell carcinoma in the hypopharyngeal region has progressive behaviour and poor prognosis. Prediction of prognosis of patients with HSCC is critical to develop novel strategy for the personalized cancer therapy. Currently, the traditional TNM system remains useful for the prediction of survival in patients with HSCC. To improve the efficiency and accuracy of the TNM system, identification of novel prognostic molecular biomarkers may help improve the prediction besides the proper combination of T, N and M stages for classifying the patients into different prognostic groups.^13^, ^14^, ^15^

miRNA has many advantages to act as biomarkers for tumour diagnosis and prognosis since it is more stable than protein, mRNA and genome in vitro, especially in clinical samples such as the FFPE tissues or other body fluids. These small nucleotides participate in and modulate the processes of cancer development, tumour invasion, metastasis and chemo/radioresistance.^16^ There is aberrantly expressed miRNA signature in head and neck carcinoma. These miRNAs could function as oncogenes or tumour suppressors in the development of human cancers. Some of these miRNAs are down‐regulated, while others are up‐regulated in SCC of head and neck (SCCHN). For example, let‐7 family was found down‐regulated in development of SCCHN, and let‐7d was associated with prognosis of SCCHN. By targeting TAGLN2,^17^ miR‐1 acted as a tumour suppressor in SCCHN.^11^ MiR‐99a was found to be down‐regulated in SCCHN, especially in SCC of oral cavity,^18^, ^19^, ^20^ subsequently contributing to the survival.^21^ In SCCHN, miR‐405 was found to inhibit tumour proliferation by targeting CDK6.^22^ Wang found miR‐203 could inhibit tumour growth and metastasis through PDPN.^23^ MiR‐15a was up‐regulated in HPV‐positive HSCC and might induce tumour apoptosis via BCL2L2 and BCL2.^24^, ^25^

However, many miRNAs are oncogenic by targeting tumour suppressor genes. miR‐21 was widely accepted as ‘oncomiR’, which was overexpressed in various types of cancers including lymphoma, oesophageal cancer, LSCC, HSCC and SCC of oral cavity.^26^, ^27^ Similarly, these oncogenic miRNAs also included miR‐16, miR‐155, miR‐130b and miR‐184.^28^, ^29^ In our current study, we found that miR‐200a‐3p, miR‐30b‐5p and miR‐4451 were up‐regulated in patients with HSCC and caused poor prognosis, indicating an oncogenic role of these miRNAs in prognosis of HSCC.

In previous studies, miR‐200a‐3p was reported to be highly expressed in several types of tumours^30^, ^31^ and led to worse prognosis,^32^ indicating that miR‐200a‐3p could be an early biomarker and a potential novel target for cancer therapeutic interventions. In vivo study, miR‐200a‐3p was found to promote cancer cell proliferation via targeting CRMP1 and inactivating tumour suppressor gene RHOA,^31^ while miR‐200a‐3p acted as a tumour suppressor gene in renal cell carcinoma or hepatocellular carcinoma.^33^, ^34^

Various studies reported the oncogenic role of miR‐30b‐5p, which was found to be up‐regulated in bladder cancer and medulloblastoma.^35^, ^36^ Shao found that miR‐30b‐5p was correlated with advanced OSCC via increasing the copy number of miR‐3b‐5p.^37^ Gaziel‐Sovran reported that miR‐30b/30d regulated the GalNAc transferase, enhancing invasion and immunosuppression of melanoma cells during metastasis.^38^ The high expression of miR‐30b‐5p was significantly associated with poor prognosis of patients with glioblastoma through mediating PRRT2.^39^ However, unlike the classic oncomiRs, miR‐30b‐5p was also reported as a tumour suppressor in oesophageal cancer, non–small‐cell lung cancer or hepatocellular carcinoma.^40^, ^41^, ^42^ Unlike miR‐200a‐3p and miR‐30b‐5p, which were widely studied in different cancers, miR‐4451 was identified more recently, for which few studies were focused on its function or mechanisms in human cancer development and prognosis. Our previous study showed miR‐4451 was highly expressed in patients with HSCC, and high expression of miR‐4451 was associated with a shorter survival of patient with HSCC,^12^ while the molecular mechanisms behind the association of miR‐4451 high expression with worse survival need more in‐depth studies.

A miRNA signature‐based method as a tool has been widely used to predict cancer risk and prognosis. As each of these miRNAs appeared to have a minor or moderate effect on OS and DSS, the combination of these miRNAs into a signature may more efficiently predict cancer outcome. Such a miRNA signature‐based classifier is of more powerful for the prediction of prognosis or early diagnosis. In our current study, we identified a novel classifier based on a 3‐miRNA signature (including miR‐200a‐3p, miR‐30b‐5p and miR‐4451), which can more accurately predict the prognosis of HSCC. The patients with high‐risk score had worse prognosis. Thus, a miRNA signature‐based method may be used to evaluate the collective effects of these miRNAs on the risk of death overall in patients with HSCC. Although significant association of these miRNAs with prognosis was found, future bioinformatics analyses or in in vivo and in vitro experiments are needed to develop further mechanisms underlying the signature.

In conclusion, our study identified a 3‐miRNA signature as potential independent prognostic predictor for patients with HSCC. Future further research and functional study are needed to explore the underlying mechanisms of these significant miRNAs in signature.

## CONFLICT OF INTEREST

None.

## AUTHOR CONTRIBUTIONS

XX and ZL performed experiments and wrote the manuscript. FZ, DL and XP assisted with the study design, data analysis and editing of the manuscript. NG and GL helped with editing of the manuscript.
